# Concurrent hollow and visceral organs traumatic injury secondary to metal shrapnel penetration in a male with situs inversus totalis: A case report

**DOI:** 10.1016/j.tcr.2022.100626

**Published:** 2022-02-21

**Authors:** Mohd Firdaus Che Ani, Muhammad Aizat Tamlikha Ismail, Nor Faezan Abdul Rashid, Firdaus Hayati

**Affiliations:** a Department of Surgery, Faculty of Medicine, Universiti Teknologi MARA, Sungai Buloh, Sungai Buloh Campus, Selayang, Selangor, Malaysia; b Department of Surgery, Selayang Hospital, Ministry of Health Malaysia, Batu Caves, Selangor, Malaysia; c Department of Surgery, Faculty of Medicine and Health Sciences, Universiti Malaysia Sabah, Kota Kinabalu, Sabah, Malaysia

**Keywords:** Congenital abnormalities, Penetrating wounds, Situs inversus, Stomach rupture

## Abstract

Situs inversus totalis (SIT) develops as a result of the embryological developmental anomaly. Managing this condition surgically is challenging as the anatomy will be mirror-imaged. A 42-year-old male had metal shrapnel broken loose from a hammer-head metal piece and pierced into his upper abdomen. A computed tomography scan of the abdomen revealed SIT with evidence of solid foreign body artefacts which were seen piercing through segment VIII of the liver and the anterior gastric wall. Exploratory laparotomy revealed a moderate amount of haemoperitoneum and a single perforation at the upper body of the stomach that was confirmed by on-table-endoscopy. The perforation was repaired with a modified Graham patch and the liver injury had stopped bleeding intraoperatively. The challenges arose during laparotomy assessment and endoscopic assessment due to inversed anatomy.

## Introduction

Surgical management of this condition can be more complicated when the anatomy is mirror-image [1]. Situs inversus is a result of the embryological developmental anomaly [2]. It is also known with other names such as situs transversus or situs oppositus [3]. It is a rare entity and only occurs in 1 in 10,000 live births [1]. It is caused by the opposite rotational direction of the viscera and organ during the organogenesis of the embryo in 3rd-week gestation [1]. It may go undiagnosed until adulthood but may be recognized by radiography as an incidental finding or during evaluation for medical assessment [3]. Situs inversus is also associated with multiple congenital anomalies such as Kartagener syndrome, atrial or ventral septal defect and horseshoe kidney which can lead to serious complications if not treated early [3], [4]. We describe a 42-year-old male with situs inversus totalis (SIT) who had a penetrating injury by a hammer-head metal piercing to the liver and discuss the challenges in managing mirror-image anatomy.

## Case report

**Fig. 1:**
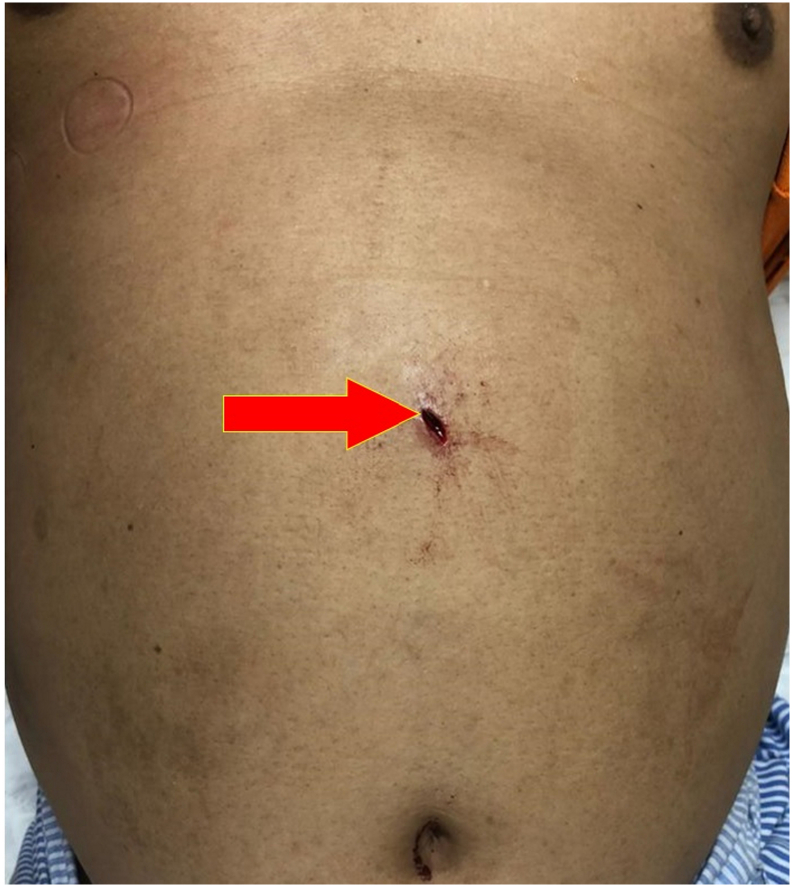
A small deep-puncture wound (red arrow) measuring 2 × 1 cm was visualized at the epigastric region.

**Fig. 2:**
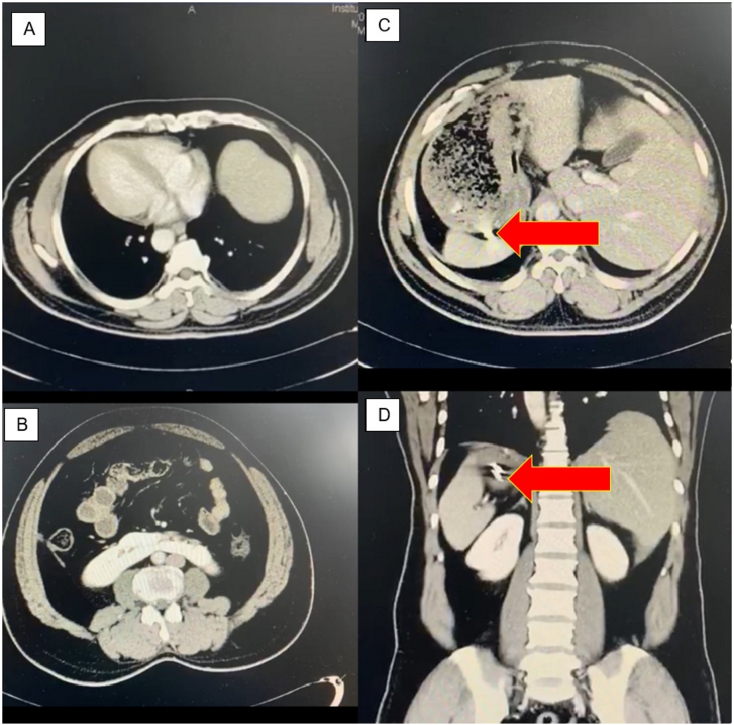
(A) A CT scan of the abdomen at the axial view showing SIT. (B) The duodenum crosses from left to right up to the duodenu-jejunal junction. (C) The foreign body artefacts (red arrow) were seen on a CT scan at the axial view. (D) The similar artefacts (red arrow) at the coronal view of the CT scan.

A 42-year-old gentleman had metal shrapnel broken loose from a hammer-head metal piece and pierced into his upper abdomen. He sustained an open wound at the upper abdomen with minimal bleeding and was brought to the Accident and Emergency Department thereafter. There was a small deep-puncture wound measuring 2 × 1 cm over the epigastric region (Fig. 1) with clots formed over the outer wound. A computed tomography (CT) scan of the abdomen revealed SIT with evidence of solid foreign-body artefacts which were seen piercing through segment VIII of the liver and the anterior gastric wall (Fig. 2).

Exploratory laparotomy was performed and intraoperatively showed 400 mls of haemoperitoneum. A single perforation was consistent with the entry wound at the upper body of the stomach measuring 1 × 1 cm with minimal gastric contamination. No exit wound was seen without an obvious foreign body found within the peritoneal cavity. On-table-endoscopy confirmed single perforation from within the stomach, but no foreign body seen. The small bowel was palpated through and a small solid object was palpable at mid-jejunum. The gastric perforation was treated as perforated gastric ulcer; perforated edges were debrided and perforation repaired with a modified Graham patch. The liver injury had stopped bleeding intraoperatively. Post-operative recovery was uneventful and he was discharged well a few days later.

## Discussion

Trauma is a pathological insult to the body. Nature, velocity, and type of injury, as well as solid versus hollow organ involvement, result in various outcomes [5], [6]. Indisputably, abnormal location can lead to diagnostic confusion and technical difficulties in surgery [1], [2]. Normal routine exposure, manoeuvre and handling of the solid organs will be tricky as these types of patients are rare in incidents. Preoperative planning with the anaesthetic team is mandatory in anticipating potential cardiac or pulmonary anomalies [1]. These associated congenital defects can lead to complex complications in polytrauma with multiple injuries [2], [4], [7]. Initial management follows a similar advanced trauma life support protocol. Penetrating injury warrants emergency exploration; via laparotomy or laparoscopy. The sequence of surgery follows the same principle of damage control surgery in trauma. A surgical exploration is warranted in cases where hollow visceral injury is evidenced via CT scan [8]. The abnormal location of intra-abdominal organs makes the identification and repair of the perforation different [7].

Foreign-body retrieval may be trickier as the object in search is only a small piece of shrapnel. Locating small objects in normal anatomy is already challenging enough, let alone in mirror-image anatomy. One can expect prolonged surgery as some adaptation is needed to identify the anatomical orientation as well as reversing the procedural steps in exchange for the left and right orientation differences [2]. Senior clinicians should be available as the vast experience of handling such cases is a huge aid in identifying the anatomy hence searching for the shrapnel will have a higher chance for success [1].

Despite mirror-image of visceral organs, the standard surgical principles and procedures apply; good access, minimal tissue dissection, good tissue handling, and good haemostasis. In our case, the patient was stable from admission, thus an abdominal CT-scan was performed to map out the foreign body, assess the extent of internal organ injury and overall plan of the procedure [1], [7]. The scanned image was useful in preparing the theatre for equipment and tools to ensure the surgery was successful. On-table endoscopy was used as an adjunct to stratify the extent of the gastric perforation to guide on delivering the best treatment for such injury. The endoscopic orientation and scope manipulation do affect the smoothness of intraoperative assessment. Following the emergency surgery, patients with SIT do not require any further intervention.

## Conclusion

SIT is a very rare entity. Trauma to these special patients is challenging in an inexperienced hand. The correct choice of investigations and comprehensive planning before surgery is vital to ensure the success of the surgery. The ability to identify and adapt to the anatomical variation is paramount; not every surgeon may have the ability to swap orientations readily. Preoperative strategy and surgical planning remain the first ideal step in such a patient.
